# Advance directives of lung cancer patients and caregivers in China: A cross sectional survey

**DOI:** 10.1111/1759-7714.13237

**Published:** 2019-12-18

**Authors:** Chenchen Feng, Juan Wu, Junying Li, Han Yu Deng, Jiewei Liu, Shuzhen Zhao

**Affiliations:** ^1^ Department of Outpatient, West China Hospital Sichuan University Chengdu China; ^2^ Cancer Center, West China Hospital Sichuan University Chengdu China; ^3^ Lung Cancer Center, West China Hospital Sichuan University Chengdu China

**Keywords:** advance directives, attitudes, caregivers, lung cancer, patients

## Abstract

**Background:**

This study aimed to investigate lung cancer patients and attitudes of their caregivers toward advance directives (ADs) in China.

**Methods:**

A cross sectional study was conducted in the Department of Oncology outpatient clinic in West China Hospital, Sichuan University. A questionnaire was used to survey the attitudes of lung cancer patients and caregivers toward ADs.

**Results:**

A total of 148 lung cancer patients and 149 caregivers were enrolled into the study. Of these, 94.6% and 89.9% of patients and caregivers had not heard of AD and none of those in the study had ever signed an AD. A total of 79.7% patients and 75.2% caregivers were willing to sign ADs after they were provided with information. Patients who preferred the end of life period to sign ADs were 5.4 times more likely to have ADs than patients who chose to sign ADs when their disease was diagnosed (*P* < 0.05, 95%CI [1.27–22.93]). Caregivers who were reluctant to undergo chemotherapy when diagnosed with cancer were 2.16 times more likely to sign ADs than those willing to receive chemotherapy (*P* < 0.05, 95%CI [1.20–3.90]).

**Conclusions:**

In China, lung cancer patients and their caregivers showed lack of knowledge about ADs, and the completion rate of ADs was extremely low. However, participants were positive about ADs and public education on ADs may help to increase the completion rate of ADs in China.

## Key points

### Significant findings of the study

Lung cancer patients and their caregivers lacked knowledge about ADs, and the completion rate of ADs was extremely low.

### What this study adds

Participants were positive about ADs and public education on ADs may help increase the completion rate of ADs in China.

## Introduction

Advance directives (ADs) are legal documents in which people choose the medical treatments they are, or are not, willing to receive if in the future they lose the capacity to talk about their wishes.^1^ It gives people the right to choose medical treatments following their values and wishes and helps to minimize suffering and maintain dignity at the last time of their life.^2^, ^3^ It has been reported that ADs are associated with a reduction in aggressive treatments,^4^, ^5^, ^6^ fewer in‐hospital deaths, increased use of hospices,^5^, ^7^ and lower hospital charges.^8^ Absence of ADs may lead to unwanted aggressive treatments,^9^ which correlates to poor quality of life.^10^ In tradition, Chinese families value interdependency between family members,^11^ and close relatives make decisions when family members lose their capacity to communicate. As a result, ADs could also help prevent family members from the difficult situation of having to guess what kind of care their loved ones would choose.

Countries such as the United States,^12^ Germany,^13^ and Singapore^14^ have already developed legislation for ADs. In 2000, Taiwan was the first place in Asia to enact legislation to provide people with the right to make do‐not‐resuscitate (DNR) decisions.^15^ However, there is no legislation for ADs in mainland China.^16^ The overall AD preparation rate is low. Previous studies have indicated that completion of ADs was 14%–30% among the general population,^17^, ^18^, ^19^ and 15%–49% among cancer patients.^18^, ^20^, ^21^, ^22^, ^23^ Many factors are associated with having ADs including sociodemographic characteristics such as age,^20^ level of education, gender, religious beliefs,^24^ health or functional status,^25^, ^26^ public education on ADs,^14^, ^27^, ^28^ and traditional perspectives.^22^ Little work has been done on the perception of ADs in both the general and ill populations. Some studies have reported the impact of patients and family members' knowledge, attitudes, and behaviors on the completion of ADs. However, most of these studies focused on various cancers^29^ or chronic diseases.^30^ Since different diseases have different diagnosis and treatment procedures and mortality differs among them, it seems necessary to focus on a specific disease. Moreover, there has been a lack of comparison between patients and caregivers since some studies have analyzed these data together.^16^, ^31^ Patients and caregivers may hold different attitudes toward ADs since healthy people may have no idea about the situation when they are ill.

Lung cancer is the most common cancer in the word, with over 1.8 million new cases and about 1.6 million deaths in 2012.^32^ It is also the leading cause of cancer‐related death.^33^ More than one third of all newly diagnosed lung cancers occur in China,^33^ with the mortality rate being higher than the international average level.^34^ To our knowledge, there has been no study about lung cancer patients’ and caregivers’ attitudes toward ADs in mainland China and also a lack of comparison between them. Therefore, we conducted this cross sectional study which aimed to investigate the attitudes of lung cancer patients and caregivers toward ADs.

## Methods

### Study design

A cross‐sectional study was conducted in the Department of Oncology outpatient clinic in West China Hospital of Sichuan University (Chengdu, China).

### Participants in the study

Between June 2017 and October 2017, 148 patients and 149 caregivers from West China Hospital, Sichuan University were enrolled in the study by convenience sampling. Patients’ inclusion criteria were: (i) Aged between 18 and 75 years old, (ii) diagnosed with lung cancer, (iii) having no communication disability with interviewers, (iv) informed consent. Exclusion criteria: (i) Do not know his or her cancer diagnosis. Caregivers inclusion criteria: (i) Aged between 18 and 75 years old, (ii) having no communication disability with interviewers, (iii) informed consent. Exclusion criteria: (i) Diagnosed with disease that may threaten their life such as cancer, heart diseases, etc.

### Measurements

Sociodemographic characteristics included gender, age, marital status, education level, relationship between caregiver and patient, address, current work status, personal income (annual), medical insurance, religion, experience in caring for seriously ill people, experiences of a loved one dying of serious illness, disease duration, Numerical Rating Scale (NRS) reflecting pain score, metastasis, complication, radiotherapy times, and cycle of chemotherapy.

Based on previous studies,^16^, ^24^, ^35^ we developed a structured questionnaire to explore the attitudes of patients and caregivers toward ADs. The content validity of the questionnaire was tested by a group of five experts in the field of end‐of‐life care and lung cancer. Items rated under four (ranged from 1 = least relevant to 5 = most relevant) were modified or deleted. After three rounds of modification, the content validity index of the questionnaire was 0.93.

### Data collection process

Patients and caregivers were informed about the aims and process of the study when they visited the Oncology outpatient clinic. After obtaining their informed consents, they were interviewed by two researchers and data were analyzed by a third researcher.

### Data analysis

SPSS software (SPSS Inc., Chicago, IL, USA; version 17.0) was used. Mean and standard deviation (SD) was used to describe continuous variables. Percentages and frequencies were applied to describe categorical variables. Chi‐square test was used to examine the proportion differences in patients and caregivers' characteristics and their attitudes toward ADs. Binary logistic regression analysis was then conducted to identify predictor of completion of ADs. *P* < 0.05 was considered statistically significant.

## Results

### Participants’ characteristics

148 lung cancer patients and 149 caregivers were enrolled into this study. The characteristics of participants are showed in Table 1. The mean (SD) age of patients and caregivers were 58.4 (11.96) and 45.18 (12.59), respectively. Most of the patients’ education level was primary and junior high school, while for caregivers, most were high school/vocational high school level. Both participants of cancer patients and caregivers mainly lived in the urban area. Characteristics including gender, age, education level, relationship between caregivers and patients, current work status, personal income, and experience in caring for seriously ill people showed a significant difference between patients and caregivers (*P* < 0.05).

**Table 1:** Characteristics of participants

Characteristics	Patients (*n* = 148)*n* %	Caregiver (*n* = 149)*n* %	*P‐*value
Gender			0.004
Female	61 (41.2)	86 (57.7)	
Male	87 (58.8)	63 (42.3)	
Age, mean	58.4 (11.96)	45.18 (12.59)	0.000
≤44	18 (12.2)	78 (52.3)	
45–59	51 (34.5)	43 (28.9)	
60–74	70 (47.3)	28 (18.8)	
≥75	9 (6.1)	0 (0)	
Marital status			0.678
Married	133 (89.9)	136 (91.3)	
Single	15 (10.1)	13 (8.7)	
Education level			0.000
Illiterate	4 (2.7)	2 (1.3)	
Primary and junior high school	82 (55.4)	33 (22.1)	
High school/vocational high school	43 (29.1)	74 (49.7)	
University and college	17 (11.5)	29 (19.5)	
Graduate school	2 (1.3)	11 (7.4)	
Relationship between caregiver and patient			0.000
Spouse	89 (60.1)	52 (34.9)	
Relatives	44 (29.8)	91 (61.1)	
Others	15 (10.1)	6 (4.0)	
Residence			0.664
Rural area	39 (26.4)	36 (24.2)	
Urban area	109 (73.6)	113 (75.8)	
Current work status			0.000
Retired	59 (39.9)	24 (16.1)	
Unemployed	46 (31.1)	46 (30.9)	
Employed	43 (29.0)	79 (53.0)	
Personal income (annual)			0.000
≤10 000	47 (31.8)	34 (22.8)	
10 000–50 000	78 (52.7)	61 (40.9)	
50 000–100 000	20 (13.5)	40 (26.8)	
≥100 000	3 (2.0)	14 (9.5)	
Medical insurance			0.304
Yes	132 (89.2)	138 (92.6)	
No	16 (10.8)	11 (7.4)	
Religiousness			0.781
Religious	8 (5.4)	7 (4.7)	
Nonreligious	140 (94.6)	142 (95.3)	
Experience in caring for seriously ill people			0.000
Yes	34 (23.0)	80 (53.7)	
No	114 (77.0)	69 (46.3)	
Disease duration			‐
<3 years	130 (87.8)	‐	
≥3 years	18 (12.2)	‐	
NRS score, median (range)	0 (0–10)	‐	‐
Metastases			‐
Yes	36 (24.3)	‐	
No	112 (75.7)	‐	
Complication			‐
Yes	28 (18.9)	‐	
No	120 (81.1)	‐	
Radiotherapy times, median (range)	0 (0–39)	‐	‐
Chemotherapy times, median (range)	0 (0–20)	‐	‐

For caregivers, disease information including disease duration, NRS score, metastases, complication, radiotherapy times, chemotherapy times were not collected.

NRS, Numerical Rating Scale (NRS) reflecting pain score.

### Perception and willingness of patients and caregivers toward ADs

A total of 140 patients (94.6%) and 134 caregivers (89.9%) had never heard of ADs. There were 118 (79.7%) patients who were willing to sign ADs when the concept of ADs was explained to them. There was a significant difference between patients and caregivers regarding the time to prepare ADs (*P* < 0.001). A total of 34.5% of patients preferred to sign ADs when treatment commenced and 31.1% chose to complete ADs when they were diagnosed with disease. A total of 112 (75.2%) caregivers were willing to sign ADs, and 42 (28.2%) caregivers chose to have ADs when they were healthy. Of the caregivers, 27.5% preferred to have ADs when they were diagnosed with incurable disease or when their incurable disease was getting worse, respectively. There was a significant difference between patients and caregivers about the question that whose advice will they largely rely on when making medical decisions (*P* < 0.001). A total of 60.2% of the patients chose medical services mainly relying on the advice of medical staff, while for caregivers, 44.3% of them would rely on themselves. As for the question when should patients know the diagnosis and prognosis of their incurable disease, patients and caregivers' attitudes showed significant differences (*P* < 0.001). A total of 64.9% of the patients chose to know about their diagnosis and prognosis soon after diagnosis, while 42.3% of the caregivers thought that patients should know their own diagnosis and prognosis and there were still 37.6% of the caregivers who preferred to conceal the diagnosis and prognosis to patients (Table 2).

**Table 2:** Perception and willingness of participants toward ADs

Variables	Patients (*n* = 148) *n* %	Caregiver (*n* = 149) *n* %	*χ* ^*2*^	*P*‐value
Heard of ADs			2.258	0.133
Yes	8 (5.4)	15 (10.1)		
No	140 (94.6)	134 (89.9)		
Attitudes toward ADs			0.885	0.347
Agree	118 (79.7)	112 (75.2)		
Disagree	30 (20.3)	37 (24.8)		
Time to prepare ADs			57.356	0.000
When healthy	0(0)	42 (28.2)		
When diagnosed with incurable disease	46 (31.1)	41 (27.5)		
When receive treatments	51 (34.5)	21 (14.0)		
When the incurable disease is getting worse	41 (27.7)	41 (27.5)		
End of life	10 (6.7)	4 (2.8)		
Making medical decisions mostly rely on whose advice			33.610	0.000
My own	28 (18.9)	66 (44.3)		
Family members	31 (20.9)	41 (27.5)		
Medical staffs	89 (60.2)	42 (28.2)		
When should patient know incurable disease diagnosis and prognosis			71.281	0.000
Do not want to know (patient should not know)	0 (0)	56 (37.6)		
Right after diagnosis	96 (64.9)	63 (42.3)		
When receiving treatment	39 (26.3)	20 (13.4)		
When the disease is getting worse	12 (8.1)	7 (4.70)		
End time of life	1 (0.7)	3 (2.0)		
Are you willing to undergo surgery? (do you want patient to undergo surgery?)			‐	0.501
Yes	145 (98.0)	143 (96.0)		
No	3 (2.0)	6 (4.0)		
If you were diagnosed with cancer one day, would you be willing to undergo surgery?				
Yes	**‐**	141 (94.6)		
No	**‐**	8 (5.4)		
Are you willing to receive radiotherapy? (do you want patient to receive radiotherapy?)			1.720	0.190
Yes	143 (96.6)	139(93.3)		
No	5 (3.4)	10 (6.7)		
If you were diagnosed with cancer one day, would you be willing to receive radiotherapy?				
Yes	**‐**	138 (92.6)		
No	**‐**	11 (7.4)		
Are you willing to undergo chemotherapy? (do you want patient to undergo chemotherapy?)			2.656	0.103
Yes	144 (97.3)	139 (93.3)		
No	4 (2.7)	10 (6.7)		
If you were diagnosed with cancer one day, would you be willing to undergo chemotherapy?				
Yes	**‐**	137 (91.9)		
No	**‐**	12 (8.1)		

Fisher's exact test was used for variable “Are you willing to undergo surgery? (do you want patient to undergo surgery?)”,so there was no *χ* ^*2*^ value for this variable.

ADs, advance directives.

The basic life‐sustaining treatment, cardiopulmonary resuscitation (CPR), and rescue medication use were the three mostly selected medical cares for all the participants at terminal and cardiac arrest status, followed by sedative drug and painkiller use (Table 3). There was no difference of medical choices at terminal status and cardiac arrest for both lung cancer patients and caregivers.

**Table 3:** Medical choices of participants at terminal status and cardiac arrest

	Terminal status		Cardiac arrest	
Medical choices	Patients %	Caregiver %	Patients %	Caregiver %
The basic life‐sustaining treatment†	134 (90.5)	137 (91.9)	136 (91.9)	123 (82.6)
CPR†	83 (56.1)	84 (56.4)	89 (60.1)	76 (51.0)
Rescue medication use (such as pressor, respiratory stimulant drugs)†	81 (54.7)	79 (53.0)	90 (60.8)	95 (63.8)
Sedative†	65 (43.9)	61 (40.9)	61 (41.2)	51(34.2)
Painkiller†	64 (43.2)	58 (38.9)	54 (36.5)	64 (43.0)
Tracheal intubation†	21 (14.2)	19 (12.8)	15 (10.1)	13 (8.7)
Stomach tube†	7 (4.7)	7 (4.7)	6 (4.0)	6 (4.0)
Urinary catheter†	7 (4.7)	8 (5.4)	6 (4.0)	6 (4.0)
Abandon†	7 (4.7)	8 (5.4)	6 (4.0)	11 (7.4)

† There was no difference for both participants’ medical choices at terminal status and cardiac arrest.

Participants could choose more than one medical choice, so the value represents how many people chose each medical care.

CPR, cardiopulmonary resuscitation.

For lung cancer patients, there were significant differences in attitude toward having ADs among patients choosing different time to have ADs (*P* < 0.01) and those relying on different people's advice to make medical choices. No differences in gender, age, marital status, education level, current work status or other characteristics were found between groups with different attitudes towards ADs (Table 4). Compared with patients who preferred to have ADs at the time of disease diagnosis, those preferring end time of life as the time to have ADs were 5.4 times more likely to have ADs in the future (Table 5).

**Table 4:** Characteristics associated with attitudes of patients toward ADs

Variables	Attitudes			
	Agree with ADs (*n* = 118)	Disagree with ADs (*n* = 30)	*χ* ^*2*^	*P*‐value
Gender, *n* (%)			1.198	0.274
Male	72 (82.8)	15 (17.2)		
Female	46 (75.4)	15 (24.6)		
Age			3.211	0.360
≤44	13 (72.2)	5 (27.8)		
45–59	39 (76.5)	12 (23.5)		
60–74	57 (81.4)	13 (18.6)		
≥75	9 (100.0)	0 (0)		
Marital status			1.089	0.297
Married	104 (78.2)	29 (21.8)		
Single	14 (93.3)	1 (6.7)		
Education level			0.903	0.924
Illiterate	3 (75)	1 (25)		
Primary and junior high school	66 (80.5)	16 (19.5)		
High school/vocational high school	33 (76.7)	10 (23.3)		
University and college	14 (82.4)	3 (17.6)		
Graduate school	2 (100)	0 (0)		
Residence			0.177	0.674
Rural area	32 (82.1)	7 (17.9)		
Urban area	86 (78.9)	23 (21.1)		
Current work status			4.509	0.105
Retired	52 (88.1)	7(11.9)		
Unemployed	35 (76.1)	11(23.9)		
Employed	31 (72.1)	12 (27.9)		
Personal income (annual)			6.485	0.090
≤10 000 RMB	35 (74.5)	12 (25.5)		
10 000–50 000 RMB	68 (87.2)	10 (12.8)		
50 000–100 000 RMB	13 (65.0)	7(35)		
≥100 000 RMB	2 (66.7)	1 (33.3)		
Medical insurance			0.240	0.625
Yes	104 (78.8)	28 (21.2)		
No	14 (87.5)	2 (12.5)		
Religiousness			0.000	1.000
Religious	6 (75)	2 (25)		
Nonreligious	112 (80)	28 (20)		
Experience in caring for seriously ill people			0.188	0.665
Yes	28 (82.4)	6 (17.6)		
No	90 (78.9)	24 (21.1)		
Disease duration				
<3 years	104 (80.0)	26 (20.0)	0.000	1
≥3 years	14 (77.8)	4 (22.2)		
NRS score			0.742	0.863
None	74 (81.3)	17 (18.7)		
Mild	26 (76.5)	8 (23.5)		
Moderate	12 (75.0)	4 (25.0)		
Severe	6 (85.7)	1 (14.3)		
Metastases			1.659	0.198
Yes	26 (72.2)	10 (27.8)		
No	92 (82.1)	20 (17.9)		
Complication			0.029	0.866
Yes	22 (78.6)	6 (21.4)		
No	96 (80.0)	24 (20.0)		
Receive radiotherapy			0.029	0.866
Yes	96 (80.0)	24 (20.0)		
No	22 (78.6)	6 (21.4)		
Receive chemotherapy			2.972	0.085
Yes	86 (83.5)	17 (16.5)		
No	32 (71.1)	13 (28.9)		
Heard of ADs			1.029	0.310
Yes	8 (100)	0 (0)		
No	110 (78.6)	30 (21.4)		
Time to prepare ADs			13.357	0.004
When healthy	0(0)	0(0)		
When diagnosed with incurable disease	36 (78.3)	10 (21.7)		
When receive treatments	46 (90.2)	5 (9.8)		
When the incurable disease is getting worse	32 (78.0)	9 (22.0)		
End time of life	4 (40.0)	6 (60.0)		
Make medical decisions mostly rely on whose advice			6.001	0.050
My own	18 (64.2)	10 (35.8)		
Family members	24 (77.4)	7 (22.6)		
Medical staff	76 (85.4)	13 (14.6)		
When should patient know incurable disease diagnosis and prognosis			3.648	0.302
Do not want to know	0 (0)	0 (0)		
Right after diagnosis	73 (76.0)	23 (24.0)		
When receiving treatment	35 (89.7)	4 (10.3)		
When the disease is getting worse	9 (75.0)	3 (25.0)		
End of life	1 (100)	0 (0)		
Are you willing to undergo surgery?			0.000	1.000
Yes	116 (80.0)	29 (20.0)		
No	2 (66.7)	1 (33.3)		
Are you willing to receive radiotherapy?			0.000	0.988
Yes	114 (79.4)	29 (20.6)		
No	4 (80.0)	1 (20.0)		
Are you willing to undergo chemotherapy?			0.054	0.816
Yes	115(79.9)	29 (20.1)		
No	3 (75.0)	1 (25.0)		

ADs, advance directives.

**Table 5:** Binary logistic regression analysis of factors which predict patients willing to prepare ADs

Variables	OR (95%CI)	*P*‐value
Timing to prepare ADs		
When diagnosed with incurable disease	1	
When receiving treatments	0.39 (0.12–1.25)	
When the disease is getting worse	1.01 (0.37–2.80)	
End of life	5.40 (1.27–22.93)	<0.05

ADs, advance directives.

For caregivers, there were differences in attitude toward having ADs among groups with different attitudes toward the management of patients, including whether patients should know the diagnosis and prognosis of the disease, willingness to advocate surgery and radiotherapy for patients, and caregivers own willingness to receive radiotherapy and chemotherapy if they were diagnosed with cancer. No differences in gender, marital status, education level, current work status or other characteristics were found between the two groups with different attitudes toward ADs (Table 6). For caregivers, those who were reluctant to undergo chemotherapy when they were diagnosed with cancer were 2.16 times more likely to prepare ADs than those willing to receive chemotherapy (Table 7).

**Table 6:** Characteristics associated with attitudes of caregivers toward ADs

Variables	Agree with ADs (*n* = 112)	Disagree with ADs (*n* = 37)	*χ* ^*2*^	*P*‐value
Gender, *n* (%)			0.061	0.805
Male	48 (76.2)	15 (23.8)		
Female	64 (74.4)	22 (25.6)		
Age			3.492	0.174
≤44	54 (69.2)	24 (30.8)		
45–59	34 (79.1)	9 (20.9)		
60–74	24 (85.7)	4 (14.3)		
Marital status			0.033	0.855
Married	103 (75.7)	33 (24.3)		
Single	9 (69.2)	4 (30.8)		
Education			1.712	0.789
Illiterate	2 (100.0)	0 (0.00)		
Primary and junior high school	26 (78.8)	7 (21.2)		
High school/vocational high school	55 (74.3)	19 (25.7)		
University and college	22 (75.9)	7 (24.1)		
Graduate school	7 (63.6)	4 (36.4)		
Residence			3.046	0.081
Rural area	31 (86.1)	5 (13.9)		
Urban area	81 (71.7)	32 (28.3)		
Current work status			2.525	0.283
Retired	20 (83.3)	4 (16.7)		
Unemployed	31 (67.4)	15 (32.6)		
Employed	61 (77.2)	18 (22.8)		
Personal income (annual)			1.970	0.579
≤10000 RMB	27 (79.4)	7 (20.6)		
10 000–50 000 RMB	43 (70.5)	18 (29.5)		
50 000–100 000 RMB	30 (75.0)	10 (25.0)		
≥100 000 RMB	12 (85.7)	2 (14.3)		
Have medical insurance			0.000	1.000
Yes	104 (75.4)	34 (24.6)		
No	8 (72.7)	3 (27.3)		
Religiousness			0.000	1.000
Religious	5 (71.4)	2 (28.6)		
Nonreligious	107 (75.4)	35 (24.6)		
Experience in caring for seriously ill people			2.161	0.142
Yes	64 (80.0)	16 (20.0)		
No	48 (69.6)	21 (30.4)		
Heard of ADs			0.569	0.440
Yes	13 (86.7)	2 (13.3)		
No	99 (73.9)	35 (26.1)		
Timing to prepare ADs			2.946	0.557
When healthy	31 (73.8)	11(26.2)		
When diagnosed with incurable disease	33 (80.5)	8 (19.5)		
When receive treatments	17 (81.0)	4 (19.0)		
When the incurable disease is getting worse	29 (70.7)	12 (29.3)		
End time of life	2 (50)	2 (50)		
Preferences for proxy			1.310	0.519
My own	47 (71.2)	19 (28.8)		
Family members	31 (75.6)	10 (24.4)		
Medical staff	34 (81.0)	8 (19.0)		
Whether the patient should know the diagnosis and prognosis of the incurable disease?			3.977	0.040
Yes	75 (80.6)	18 (19.4)		
No	37 (66.1)	19 (33.9)		
When should patient know incurable disease diagnosis and prognosis			7.222	0.100
Patient should not know	37 (66.1)	19 (33.9)		
Right after diagnosis	53 (84.1)	10 (15.9)		
When receiving treatment	16 (80.0)	4 (20.0)		
When the disease is getting worse	4 (57.1)	3(42.9)		
End time of life	2 (66.7)	1(33.3)		
Do you want patient to undergo surgery?			3.759	0.050
Yes	110 (76.9)	33 (23.1)		
No	2 (33.3)	4 (66.7)		
If you were diagnosed with cancer one day, would you be willing to undergo surgery?			1.621	0.203
Yes	108 (76.6)	33 (23.4)		
No	4 (50.0)	4 (50.0)		
Do you want patient to receive radiotherapy?			5.227	0.020
Yes	108 (77.7)	31 (22.3)		
No	4 (40.0)	6 (60.0)		
If you were diagnosed with cancer one day, would you be willing to receive radiotherapy?			4.837	0.030
Yes	107 (77.5)	31(22.5)		
No	5 (45.5)	6 (54.5)		
Do you want patient to undergo chemotherapy?			3.170	0.080
Yes	107 (77.0)	32 (23.0)		
No	5 (50.0)	5 (50.0)		
If you were diagnosed with cancer one day, would you be willing to undergo chemotherapy?			3.870	0.040
Yes	106 (77.3)	31 (22.7)		
No	6 (50.0)	6 (50.0)		

ADs, Advance directives.

**Table 7:** Binary logistic regression analysis of factors which predict caregivers willing to prepare ADs

Variables	OR (95%CI)	*P*‐value
If you were diagnosed with cancer one day, would you be willing to undergo chemotherapy?		
Yes	1	
No	2.16(1.20–3.90)	<0.05

CI, confidence interval; OR, Odds Ratio.

## Discussion

To the best of our knowledge, this is the first study to focus on lung cancer patients' and caregivers' attitudes toward ADs in China. In our study, 94.6% of the patients and 89.9% caregivers had never heard of ADs, and none had signed ADs. Our results were similar to a previous study conducted in China focusing on various cancers,^24^ in which all of the 526 cancer patients did not have ADs, and 90% of them had never heard of the terminology of “advance directive”. There were obvious disparities in the comparison of the rate of completion of ADs in China with that of other countries that had AD legislation. In Germany, 18% of cancer patients, 19% of healthy people,^18^ and 28%–31% hematology and oncology outpatient patients had signed ADs.^36^, ^37^ In Korea, 35.5% of patients in a Hospice Center completed ADs,^23^ and in the report by White *et al*. 14% of the Australian population had ADs.^17^ After being informed about the concept of ADs, 79.7% of the patients and 75.2% of the caregivers were willing to have ADs, which was in line with previous studies conducted in various cancer patients.^16^ Interestingly, at the very beginning, our researchers were concerned about the cancer patients’ cooperation with the investigation. However, during the investigation, most of the patients were cooperative, indicating their willingness to accept ADs. China has experienced great development and fast modernization during the past several decades, and the behavior of Chinese people and their values have greatly changed with the rapid development of the economy.^38^ At the same time, patients’ sense of autonomy and self‐determination has increased,^16^ which may help explain the overall positive attitude toward ADs. A previous report indicated that insufficient knowledge was frequently given as the reason for declining ADs.^36^ Therefore, it reminds us that the legislation of ADs should be placed on the agenda, and that wider public education of the benefit of ADs is essential.

We also found some interesting differences of participants’ attitude toward several questions. Most patients made their decisions mainly relying on the information and advice of medical staff. However, for caregivers, the decisions they made were self‐reliant. This may be explained by when people are ill, they may want a cure for their diseases or relief of their symptoms so they must rely on medical staff, while healthy people cannot put themselves into a situation where they are ill, and they don't have to rely on the advice of medical staff. Therefore, they may think that their own willingness is more important, which demonstrates that different attitudes toward ADs are dependent upon health status. Therefore, we need to investigate the attitude of people towards ADs when they are both healthy and ill.

In China, many caregivers would not tell patients about their disease diagnosis and prognosis for the fear of the pressure this may place on them. However, the results of our study indicated that all patients wanted to know their diagnosis and prognosis, but there were still 37.6% of caregivers who said that they did not want patients to know the truth. The differences between patients and caregivers toward disease, life, and death should be given more attention. In Chinese culture, when people lose their ability to communicate, caregivers play an important role in assisting patients to choose the medical options available to them. To be aware of a person's attitude and willingness when they are healthy may assist caregivers in making decisions for patients in the future.

Patients and caregivers were invited to choose their medical cares when they were at terminal stage and cardiac arrest. Before that, the meaning of terminal status and cardiac arrest was explained to them. Basic life‐sustaining treatment, CPR, and use of rescue medication were the main options chosen by participants of both health statuses, followed by use of sedatives and painkillers. Other invasive procedures such as tracheal intubation, or use of a stomach tube were less preferred. There were no significant differences of willingness to medical treatments between lung cancer patients and caregivers and both patients and healthy people showed their first choice was to receive life saving treatments. Our results were different to those of the study of Zheng *et al*.^24^ in which the majority of patients chose comfort care only, followed by limited care. The reasons may be that 82.1% of the participants in the report by Zheng *et al*. were religious and the study reported that faith can influence cancer patients' medical decisions, and make them more likely to refuse the recommendations of physicians.^39^ Moreover, the study by Zheng *et al*. included patients with various cancers, while our study only focused on lung cancer patients. The survival or mortality rate can be different in a wide range of diseases, and therefore patients may have different confidence in overcoming the disease. Furthermore, contrary to our study, in the study by Kong *et al*.^23^ most terminal cancer patients did not want to receive any invasive medical interventions to sustain life. Possible reasons for this may be that our patients were not yet at the terminal stage of illness and terminal cancer patients may already have suffered many invasive treatments and have known about their prognosis. This indicates that we should pay attention to the possibility of patients wanting to change their end of life AD and future studies should be conducted which take this preference for care into account.

Patients preferred end time of life as the time when an AD should be in place and were 5.4 times more likely to have ADs at that stage than those choosing to have ADs when first diagnosed. A previous study has reported that completion of ADs in the last months of life was associated with higher rates of aggressive care preferences.^26^ Allison *et al*. also reported that those preparing too late may be in a rush to make a decision and did not represent patients' values and willingness.^40^ Those results confirm that attention should be paid to those who prepare ADs at the end time of life because ADs may cannot help to maintain people's dignity at that time. For those patients, early education so that they understand the meaning of ADs should be compulsory.

For caregivers, those who were reluctant to undergo chemotherapy when they become ill were 2.16 times more likely to prepare ADs than those willing to receive chemotherapy. Chemotherapy is a commonly used treatment in the majority of lung cancer patients, which might prolong survival but can still cause adverse effects.^9^ This result may be because some people are afraid of the adverse effects of treatment and the suffering which can be caused by chemotherapy, and those choosing not to receive chemotherapy showed that they want more comfortable treatments, but not to prolong survival. Therefore, they would like to have ADs to reflect their willingness.

Our study had some limitations. First, it was conducted only in one single hospital and the sample size was small both for patients and caregivers, which may be the reason why the study has not highlighted other factors. Multi‐center studies with a larger sample size are required in the future to improve the representativeness of the participants. Second, some important information such as patients'cancer stage could not be accessed due to the cross sectional study design. As a result, some meaningful results may have been left out of the investigation.

In conclusion, Chinese lung cancer patients and their caregivers lacked knowledge concerning ADs, and the completion rate of ADs was extremely low. However, after being informed about them, most indicated that they would be willing to prepare ADs. Public education on ADs is necessary for Chinese people, and may help increase the completion rate of ADs in China. Patients preferred end of life as the time to have ADs were more likely to prepare ADs, but attention should be paid because their medical decisions at that time may be made in haste and cannot reflect their true willingness to participate. For those patients, early education on ADs may help them express their preferences. Caregivers who were reluctant to undergo chemotherapy if they became ill one day were more inclined to prepare ADs than those willing to receive chemotherapy.

## Disclosure

The authors have nothing to declare.
